# A retrospective analysis of the anatomic characteristics of pulmonary bullae based on chest CT classification and their association with pulmonary function

**DOI:** 10.3389/fmed.2026.1752828

**Published:** 2026-04-29

**Authors:** Xin Zou, Xiaoqing Zhou, Weixin Zou, Xiaoxia Chen, Lei Pan, Wangping Li

**Affiliations:** 1 Department of Respiratory and Critical Care Medicine, Tangdu Hospital, Air Force Medical University, Xi’an, Shaanxi, China; 2 Department of Tuberculosis, The 987th Hospital of Joint Logistic Support Force, Baoji, Shaanxi, China; 3 Department of Disease Control and Prevention, Xi’an Eighth Hospital, Xi’an, Shaanxi, China; 4 Department of Graduate Studies, Xi’an Medical University, Xi’an, Shaanxi, China

**Keywords:** bullous emphysema, computed tomography, COPD—chronic obstructive pulmonary disease, pulmonary bullae, pulmonaryfunction assessment

## Abstract

**Objective:**

To propose a refined CT-based phenotypic classification of pulmonary bullae and investigate the differences in demographic characteristics, morphological features, and pulmonary function among different subtypes.

**Methods:**

This retrospective study enrolled 467 patients diagnosed with pulmonary bullae who underwent chest CT at Tangdu Hospital from January 2023 to December 2024. Patients were classified into three phenotypes: bullous emphysema, intrapulmonary bullae, and subpleural bullae. Demographic data, smoking history, BMI, bulla morphology (size, number, internal septations, wall thickness, margin regularity), and pulmonary function parameters (FVC, FEV_1_, FEV_1_/FVC, DLCO) were collected and compared among groups. Subgroup analyses were performed based on smoking status, BMI level, and COPD status.

**Results:**

Significant intergroup differences were observed in age, BMI, and smoking history (*P* < 0.05). Patients with bullous emphysema were older, had lower BMI, and higher smoking rates. Morphologically, the bullous emphysema group exhibited significantly greater maximum bulla length, width, and surface area compared to the other two groups (*P* < 0.05). Smoking was associated with larger bullae, higher proportions of internal septations and thick walls, and fewer regular margins (*P* < 0.05). Low BMI patients (BMI < 23.03 kg/m^2^) demonstrated larger bullae with more complex morphology compared to high BMI patients (*P* < 0.05). Functionally, the bullous emphysema group had significantly lower FEV_1_, FEV_1_% predicted, FEV_1_/FVC, and DLCO% predicted (*P* < 0.05). Patients with COPD (*n* = 83) showed larger bullae and lower proportions of regular margins and internal septations compared to non-COPD patients (*P* < 0.05).

**Conclusion:**

CT-based phenotyping of pulmonary bullae reveals distinct demographic, morphological, and functional profiles. Bullous emphysema is associated with more severe morphological abnormalities and pulmonary function impairment. Smoking and low BMI are modifiable factors promoting bulla progression. This classification provides a foundation for personalized clinical surveillance and intervention strategies.

## 1 Introduction

Pulmonary bullae are defined as air-filled cystic spaces exceeding 1 cm in diameter, representing the end-stage outcome of alveolar destruction and coalescence resulting from various pathological insults, such as infection, chronic inflammation, or emphysema (1). As a common structural lung disease, pulmonary bullae not only constitute the primary etiology of spontaneous pneumothorax (2, 3) but also represent a significant imaging phenotype in chronic obstructive pulmonary disease (COPD) (4). Their presence is closely associated with declined pulmonary function, impaired quality of life, and adverse prognosis (4).

In clinical practice, chest computed tomography (CT) serves as the essential modality for the identification and evaluation of pulmonary bullae. CT imaging not only confirms the presence of lesions but also provides detailed morphological characterization, including shape (round, oval, or irregular), wall thickness, the presence of internal septations, distribution pattern (solitary or diffuse), number, size, and spatial anatomical relationship with the pleura and adjacent lung parenchyma. Based on these morphological features, pulmonary bullae are commonly classified into three main types in clinical settings (4–6): bullous emphysema, intrapleural pulmonary bullae, and subpleural pulmonary bullae. This classification extends beyond mere description; the nomenclature directly reflects both the coexistence with emphysema and the anatomical relationship with the pleura, with distinct morphological profiles corresponding to different pathophysiological mechanisms.

Current understanding and research on pulmonary bullae exhibit significant limitations. There is a notable lack of systematic, quantitative comparisons of morphological parameters—such as precise size measurements, qualitative analysis of wall thickness, and specific distribution patterns—among different bullae phenotypes. This absence of detailed characterization hinders an in-depth radiological differentiation of the fundamental differences between bullae types. Consequently, current clinical assessment frameworks often lack the precision and individualization necessary to reliably predict the risk of pulmonary function impairment and disease progression trajectories in individual patients.

To address this gap, the present study retrospectively enrolled 467 patients with pulmonary bullae for systematic analysis. Based on chest CT findings, patients were categorized into three phenotypic groups: bullous emphysema, intrapulmonary bullae, and subpleural bullae. Subsequently, the morphological characteristics of the bullae in each group were meticulously described and quantitatively compared, and their associations with pulmonary function test results were analyzed. Through this approach, we aim to preliminarily establish an imaging-pulmonary function profile for distinct pulmonary bullae phenotypes, thereby providing empirical evidence to elucidate the heterogeneity of pulmonary bullae and facilitate more precise disease assessment.

## 2 Materials and methods

### 2.1 Patient data collection

Patients who underwent chest computed tomography (CT) examinations at Tangdu Hospital between January 1, 2023, and December 31, 2024, were included in this study. From the radiology reports, patient IDs with a diagnosis of “pulmonary bullae” or “pulmonary bulla” were identified, resulting in a total of 467 patients with pulmonary bullae being selected for analysis. Smoking history was defined as encompassing both current and former smokers. Body Mass Index (BMI) is calculated as an individual’s weight in kilograms divided by the square of their height in meters.

**FIGURE 1:**
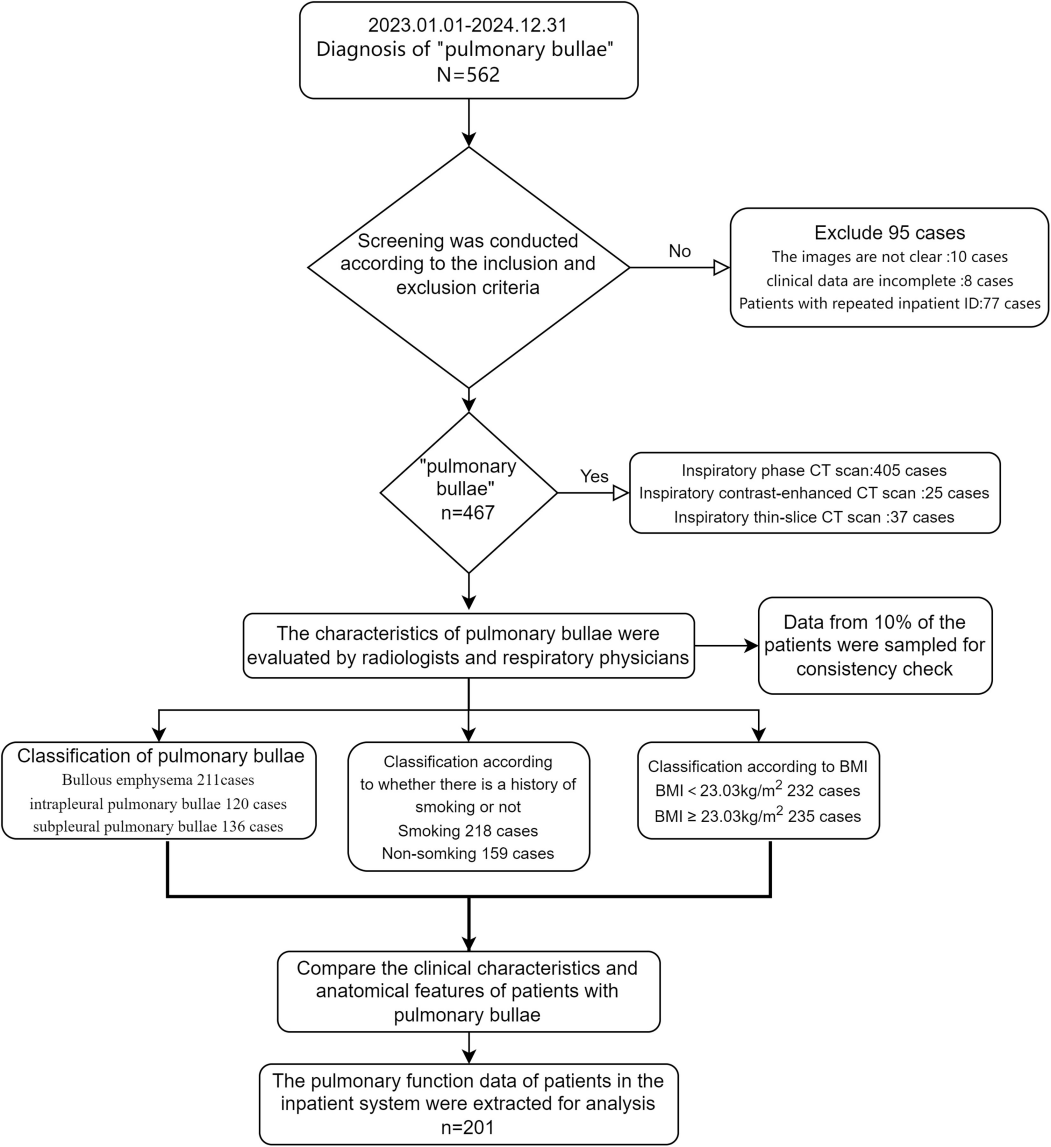
Research flowchart.

The image observation and measurement software used in this study was Mi-platform (version 1.3.8.2689), developed by Hinacom Software and Technology, Ltd. (Beijing). This software enables the measurement of pulmonary bullae size and allows continuous observation of both the lung window and mediastinal window in chest CT scans. CT Data Acquisition Parameters: As a retrospective study, all chest CT images in this research were acquired using a GE Revolution CT scanner. The scanning parameters were as follows: Tube Voltage: 100–120 kVp; Tube Current: 30–200 mAs; Detector Collimation: 128 × 0.6 mm; Pitch: 1.2, 0.98; Slice Thickness: 5 mm; Reconstruction Algorithm: Images were reconstructed using a standard kernel (Kernel/B) and/or a high-resolution kernel (Kernel/C); Reconstruction Slice Thickness: 1.0 mm; Matrix: 512 × 512; Scan Range: The entire lung was covered from the lung apex to the costophrenic angle. Scan Mode: All scans were performed at full inspiration with breath-holding. All examinations adhered to our institution’s standard low-dose chest CT protocol, which aims to minimize radiation exposure while maintaining diagnostic image quality. All images were independently reviewed by experienced attending physicians (with more than 5 years of practice) from both the respiratory medicine and radiology departments. To assess inter-observer reliability, a randomly selected 5% of the study sample was re-evaluated by the same observers. Both the Intraclass Correlation Coefficient (ICC) and Kappa statistic exceeded 0.75, indicating good reproducibility. The research flow diagram of this study is shown in Figure 1.

#### 2.1.1 Inclusion criteria

(1) Clinical indications for CT examination included, but were not limited to, respiratory symptoms (e.g., cough, chest pain, dyspnea), abnormal pulmonary findings on physical examination, or follow-up of pre-existing pulmonary diseases;
(2) CT findings met the diagnostic criteria for pulmonary bullae, defined as air-filled cystic spaces with a diameter greater than 1 cm (1);
(3) CT images were complete and of sufficient clarity for analysis;
(4) The diagnostic process for pulmonary bullae followed the stepwise diagnostic approach for cystic lung diseases (7);
(5) Patients were aged ≥ 18 years;
(6) Predominantly Han Chinese population from the Northwest China region

#### 2.1.2 Exclusion criteria

Patients who met the above inclusion criteria but presented with any of the following conditions were excluded from the study:

(1) Coexisting congenital pulmonary bullae or other congenital cystic lung developmental abnormalities;
(2) Presence of other cystic lung lesions on chest CT, specifically including: pulmonary cysts (round low-attenuation areas with a clear interface with normal lung, typically air-containing, thin-walled with wall thickness < 2 mm, and usually not associated with emphysema); paraseptal emphysema (subpleural and peribronchovascular low-attenuation areas separated by intact interlobular septa, sometimes associated with bullae, without distinct walls); honeycombing (closely approximated ring-like shadows with diameters of 3–10 mm, typically subpleural, with well-defined walls of variable thickness ranging from 1 to 3 mm, mostly accompanied by other signs of fibrosis) (8);
(3) Poor CT image quality or missing information precluding accurate evaluation;
(4) Incomplete baseline clinical data that could affect the study analysis.
(5) Patients with a time interval exceeding 1 month between chest CT examination and pulmonary function testing were excluded.

This retrospective study adhered to the principles of the Declaration of Helsinki and was approved by the Medical Ethics Committee of the Tangdu Hospital of Air Force Medical University (Ethics Approval No. K202503-02).

### 2.2 Baseline patient characteristics

This study included a total of 467 patients with pulmonary bullae. Among them, 388 (83.1%) were male and 79 (16.9%) were female. The age range of the patients was 56–71 years. Mean body mass index (BMI) was 23.0 ± 3.41 kg/m^2^. In terms of smoking history, 218 patients (46.7%) had a history of smoking, 159 patients (34.0%) were non-smokers, and the smoking history was unavailable for 90 patients (19.3%). Among the 275 patients with specified disease histories, the pulmonary comorbidities* of pulmonary bullae were primarily emphysema (56.00%), pulmonary nodules (39.27%), and pulmonary infection (21.82%). The extrapulmonary comorbidities* were mainly hypertension (36.3%), coronary heart disease (27.27%), and diabetes mellitus (18.55%). Detailed explanations of pulmonary bullae and other key terms are provided in Table 1. The characteristics of patients with pulmonary bullae are shown in Table 2.

**TABLE 1:** Definitions and explanations of key entries.

Entry	Definitions and explanations
Bullous emphysema. Chest CT findings are shown in Figure 2A.	On chest CT, the lesion manifests as a well-defined, round focal area with increased lucency or decreased attenuation, surrounded by a thin wall measuring less than 1 mm in thickness. This finding is commonly associated with adjacent emphysematous changes and may present with panlobular emphysema involving all pulmonary lobules (4).
Intrapleural pulmonary bullae. Chest CT findings are shown in Figure 2B.	The cavity, located within the visceral pleura, exhibits a well-defined boundary with the adjacent alveoli. Its surface is lined by a thin layer of visceral pleura, while its base comprises normal alveolar tissue (5, 6).
Subpleural pulmonary bullae. Chest CT findings are shown in Figure 2C.	Situated subjacent to the visceral pleura, this lesion arises from the destruction of pulmonary parenchyma and commonly occurs as a secondary manifestation of emphysema (5, 6).
Pulmonary bullae demonstrate well-defined and regular morphological features	Morphological symmetry: Characterized by approximately circular or elliptical configurations.
The walls of pulmonary bullae are smooth	Marginal contour: The boundaries are well-defined and smooth, with no evident lobulation, notching, or spiculation. Additionally, there are no fibrous bands interdigitating with the surrounding lung parenchyma or signs of inflammatory infiltration.
Intrapulmonary bullae contain septa, leading to the formation of multilocular configurations	Within a solitary pulmonary bulla, fibrous or pulmonary parenchymal septa may be observed, subdividing the cystic cavity into multiple locules that are either interconnected or discrete.
Thick-walled pulmonary bullae	The thickness of the cyst wall ranges from 1 mm to 4 mm (8).
Parenchymal opacification, manifesting as consolidation or ground-glass opacities, may be observed in the lung lobe harboring pulmonary bullae	Consolidation indicates that definition of these margins (excepting air bronchograms) is lost within the dense opacification, whereas ground-glass opacity indicates a smaller increase in attenuation, in which the definition of underlying structures is preserved (9).

**FIGURE 2:**
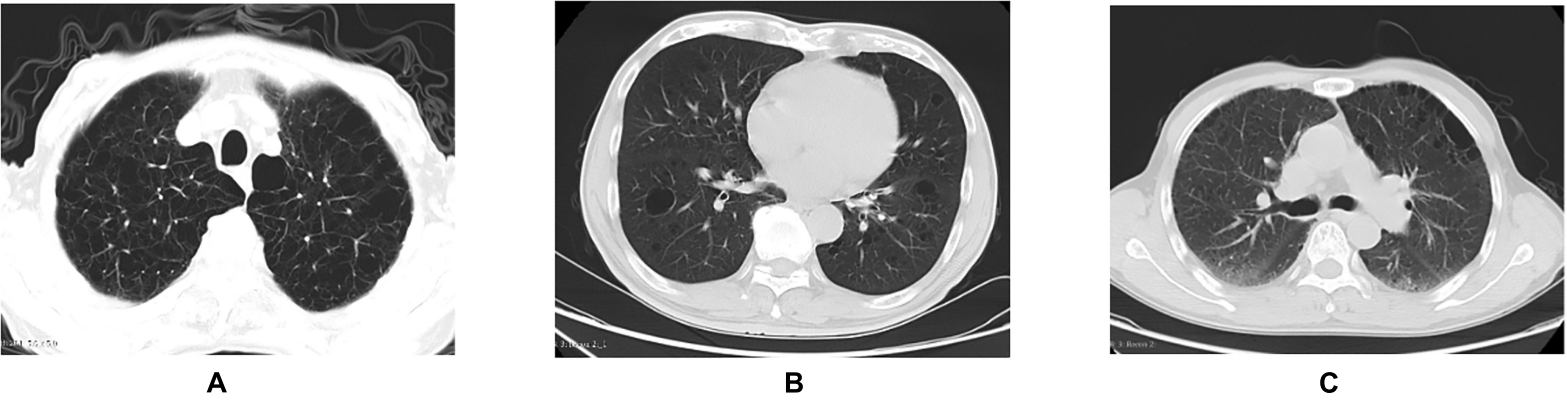
From left to right **(A–C)**. **(A)** Bullous emphysema (Male, 81 years old; pulmonary function tests: FEV_1_/FVC = 60.36%, FEV_1_%pred = 26.21%; indicative of very severe obstructive ventilatory impairment). **(B)** Intrapleural pulmonary bullae (Male, 68 years old, non-smoker; pulmonary function tests: mild obstructive ventilatory impairment). **(C)** Subpleural pulmonary bullae (Male, 61 years old; pulmonary function tests: mild obstructive ventilatory impairment).

**TABLE 2:** Demographic characteristics and features of pulmonary bullae in patients.

Characteristics	n%/mean ± SD/ Median[IQR]
Gender	
Male	388(83.1%)
Female	79(16.9%)
Age (years)	64(56–71)
BMI (kg/cm^2^)	23.0 ± 3.41
Smoking history	
Yes	218(46.7%)
No	159(34.0%)
Unknow	90(19.3%)
Classification of pulmonary bullae	
Bullous emphysema	211(45.2%)
Intrapleural pulmonary bullae	120(25.7%)
Subpleural pulmonary bullae	136(29.1%)
Number of pulmonary bullae	
1	142(30.4%)
2–5	50(10.7%)
>5	275(58.9%)
Comorbidities specified	
Yes	275
No	192
Pulmonary comorbidities (n = 275)*	
Emphysema	154(56.0%)
Pulmonary nodules	108(39.27%)
Pulmonary infection	60(21.82%)
Pulmonary interstitial fibrosis	41(14.91%)
Extrapulmonary comorbidities (n = 275)*	
Hypertension	100(36.3%)
Coronary heart disease	75(27.27%)
Diabetes mellitus	51(18.55%)

*Since some patients presented with multiple underlying diseases, the sum of the cases for each comorbidity category exceeds the total number of cases, and the sum of the percentages exceeds 100%.

### 2.3 Statistics

Statistical analyses were performed using SPSS software (Version 26.0, IBM Corp., Armonk, NY, United States). Continuous variables were first assessed for normality using the Kolmogorov-Smirnov test. Variables with a normal distribution (*P* > 0.05) were expressed as mean ± standard deviation (mean ± SD), while non-normally distributed variables were presented as median (interquartile range) [M (Q1, Q3)]. Categorical variables were expressed as frequencies (percentages) [n (%)].

Comparisons between groups were performed as follows:

(1) Categorical variables: Comparisons between two groups were conducted using the chi-square test (when expected frequencies were ≥ 5). For comparisons among multiple groups, either one-way ANOVA or the Kruskal-Wallis H test was used, depending on the data distribution and homogeneity of variances. Homogeneity of variances was assessed using Levene’s test (*P* > 0.05 indicating homogeneity).
(2) Continuous variables: Prior to group comparisons, Levene’s test was performed to assess homogeneity of variances (*P* > 0.05 indicating homogeneity). For comparisons between two groups, the independent samples *t*-test was used for data that were normally distributed with homogeneous variances; otherwise, the Mann-Whitney U test was applied. For comparisons among multiple groups, one-way ANOVA was used for normally distributed data, with *post hoc* pairwise comparisons conducted using the LSD-*t*-test; for non-normally distributed data, the Kruskal-Wallis H test was employed.

## 3 Results

### 3.1 Comparison of imaging characteristics among different CT-defined phenotypes of pulmonary bullae

**FIGURE 3:**
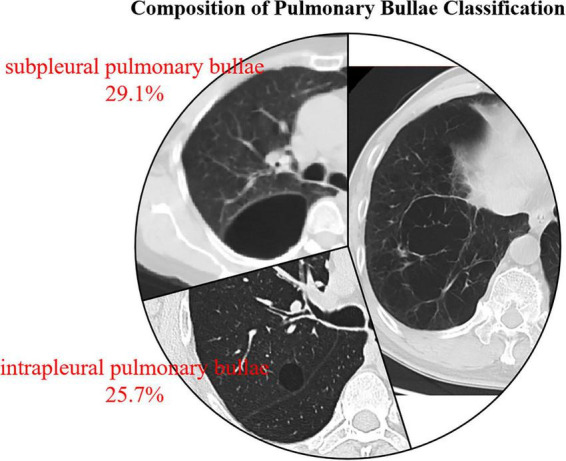
Proportions of different types of pulmonary bullae (created by PowerPoint, Chest CT images of pulmonary bullae were obtained from patients in this study with informed consent.)

**FIGURE 4:**
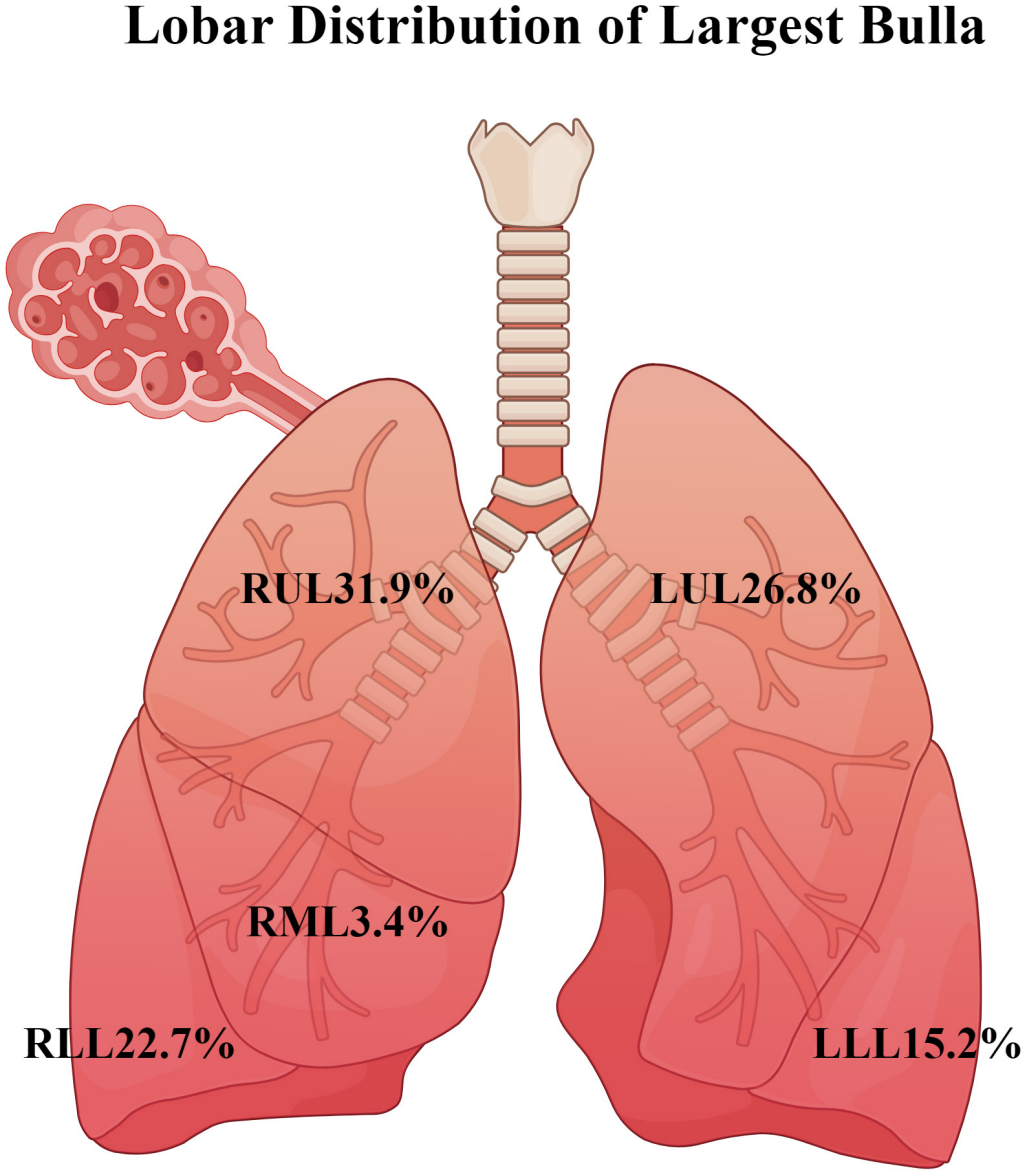
Proportional distribution of the largest pulmonary bulla by pulmonary lobe (created by figdraw.com).

The demographic information and characteristics of pulmonary bullae were analyzed in three groups of patients: those with bullous emphysema, intrapleural pulmonary bullae, and subpleural pulmonary bullae. The detailed comparisons are presented in Table 3. Significant differences were observed among the three groups in maximum bulla length, maximum bulla width, and surface area using the Kruskal-Wallis H test (*P* < 0.05). Subsequent pairwise comparisons using the Bonferroni-corrected Mann-Whitney U test revealed that the bullous emphysema group had significantly greater maximum bulla length, maximum bulla width, and surface area compared to the other two groups (*P* < 0.05). Additionally, the maximum bulla length in the subpleural pulmonary bullae group was significantly greater than that in the intrapleural pulmonary bullae group (*P* < 0.05). The proportions of the three groups of bulla types are shown in Figure 3. The distribution of the largest bullae across different lung lobes is shown in Figure 4.

**TABLE 3:** Comparison of demographic features and characteristics of pulmonary bullae among patients with different pulmonary bullae types.

Characteristics		Classification			*F*/χ^2^/*H*	*P*
		Bullous emphysema (n = 211)	Intrapleural pulmonary bullae(n = 120)	Subpleural pulmonary bullae (*n* = 136)		
Maximum bulla size	Long diameter (mm)	30.70(21.80–45.22)	14.50(12.00–19.15)^b^	19.25(15.70–26.48)^bc^	148.484	<0.001
	Short diameter (mm)	19.60(15.05–30.05)	11.45(9.25–16.00)^b^	12.65(10.12–17.52)^b^	111.356	< 0.001
	Surface area (mm^2^)	468.59(267.09–1073.19)	132.12(84.28–234.81)^b^	185.84(127.93–341.89)^bc^	139.090	< 0.001
Distribution of the largest pulmonary bullae	RUL	83(39.34%)	22(18.33%)	44(32.35%)	47.750	<0.001
	RML	1(0.47%)	9(7.50%)	6(4.41%)		
	RLL	40(18.96%)	42(35.00%)	24(17.65%)		
	LUL	57(27.01%)	20(16.67%)	48(35.29%)		
	LLL	30(14.22%)	27(22.50%)	14(10.29%)		
Number of pulmonary bullae	1	5(2.37%)	81(67.50%)	56(41.18%)	237.043	<0.001
	2–5	4(1.90%)	19(15.83%)	27(19.85%)		
	>5	202(95.73%)	20(16.67%)	53(38.97%)		
Regular edges	Yes	44(20.85%)	101(84.17%)	66(48.53%)	124.674	<0.001
	No	167(79.15%)	19(15.83%)	70(51.47%)		
Edges smooth	Yes	138(65.40%)	111(92.50%)	103(75.74%)	30.274	<0.001
	No	73(34.60%)	9(7.50%)	33(24.26%)		
Presence or absence of septa within the bullae	Yes	188(89.10%)	29(24.17%)	79(58.09%)	141.285	<0.001
	No	23(10.90%)	91(75.83%)	57(41.91%)		
Presence of thick-walled bullae (wall thickness > 1mm)	Yes	118(55.92%)	31(25.83%)	72(52.94%)	30.214	<0.001
	No	93(44.08%)	89(74.17%)	64(47.06%)		
Whether there is infiltration in the lobe where the bulla is located	Yes	89(42.18%)	26(21.67%)	48(35.29%)	14.180	0.001
	No	122(57.82%)	94(78.33%)	88(64.71%)		

^a^Compared with bullous emphysema, indicates *P*^a^ < 0.05 after intergroup LSD-t test.

^b^Compared with bullous emphysema, indicates *P*^b^ < 0.05 after intergroup Mann-Whitney U test with Bonferroni correction.

^c^Compared with intrapleural pulmonary bullae, indicates *P*^c^ < 0.05 after intergroup Mann-Whitney U test with Bonferroni correction. Bullae surface area (mm^2^) = π/4 × Long diameter × Short diameter. RUL, Right Upper Lobe of the lung; RML, Right Middle Lobe of the lung; RLL, Right Lower Lobe of the lung; LUL, Left Upper Lobe of the lung; LLL, Left Lower Lobe of the lung; F, ANOVA statistic; χ^2^, Chi-square value; H, Kruskal-Wallis H statistic.

### 3.2 The correlation between smoking history and pulmonary bullae

A total of 377 patients with documented smoking history were included in the analysis and were divided into two groups based on smoking status to evaluate the impact of smoking on pulmonary bullae.

As shown in Table 4, significant differences were observed in gender distribution between the two groups, with males predominating in the smoking group (*P* < 0.05). No statistically significant differences were found in age or BMI between the two groups (*P* > 0.05). Significant differences were observed in the distribution of pulmonary bullae phenotypes and the location of maximum bulla size between the two groups (*P* < 0.05).

**TABLE 4:** Comparison of the characteristics of pulmonary bullae in patients with or without a history of smoking.

Characteristics	Smoking		χ^2^/F/U	*P*
	Smoking (*n* = 218)	Non-smoking (*n* = 159)		
Gender			104.479	< 0.001
Male	216(99.08%)	92(57.86%)		
Female	2(0.92%)	67(42.14%)		
Age (years)	66(59–71)	64(56–72)	−0.794	0.427
BMI (kg/m^2^)	22.48 ± 3.26	23.74 ± 3.44	0.094	0.759
Classification			61.523	< 0.001
Bullous emphysema	128(58.72%)	37(23.27%)		
Intrapleural pulmonary bullae	30(13.76%)	73(45.91%)		
Subpleural pulmonary bullae	60(27.52%)	49(30.82%)		
Maximum bulla size				
Long diameter (mm)	24.80(18.30–41.65)	16.60(12.5–26.40)	−6.541	< 0.001
Short diameter (mm)	16.90(12.40–23.05)	12.50(10.10–19.00)	−4.694	< 0.001
Surface area (mm^2^)	310.69(171.59–754.99)	165.88(99.91–371.38)	−5.839	< 0.001
Distribution of the largest pulmonary bullae			21.620	< 0.001
RUL	84(38.53%)	34(21.38%)		
RML	4(1.83%)	11(6.92%)		
RLL	42(19.27%)	48(30.19%)		
LUL	60(27.52%)	37(23.27%)		
LLL	28(12.85%)	29(18.24%)		
Number of pulmonary bullae			79.440	< 0.001
1	34(15.60%)	91(57.23%)		
2–5	22(10.09%)	19(11.95%)		
>5	162(74.31%)	49(30.82%)		
Regular edges			36.521	< 0.001
Yes	74(33.94%)	104(65.41%)		
No	144(66.06%)	55(34.59%)		
Edges smooth			6.584	0.011
Yes	153(70.18%)	130(81.76%)		
No	65(29.82%)	29(18.24%)		
Presence or absence of septa within the bullae			71.689	< 0.001
Yes	171(78.44%)	56(35.22%)		
No	47(21.56%)	103(64.78%)		
Presence of thick-walled bullae (wall thickness > 1mm)			8.373	0.004
Yes	108(49.54%)	55(34.59%)		
No	110(50.46%)	104(65.41%)		

F, ANOVA statistic; χ^2^, Chi-square value; U, Mann-Whitney U test.

The smoking group demonstrated significantly greater maximum bulla length, maximum bulla width, and surface area compared to the non-smoking group (*P* < 0.05).

The proportion of patients with “internal septations” and “thick-walled” bullae was significantly higher in the smoking group than in the non-smoking group (*P* < 0.05).

The proportion of patients with “regular margins” and “smooth borders” was significantly higher in the non-smoking group compared to the smoking group (*P* < 0.05).

### 3.3 Comparison of pulmonary bullae characteristics in patients of different body types

To analyze the characteristics of pulmonary bullae in patients with different BMI levels, patients were categorized into two groups based on the median BMI of 23.03 kg/m^2^: low BMI (BMI < 23.03 kg/m^2^) and high BMI (BMI ≥ 23.03 kg/m^2^). The characteristics of pulmonary bullae were compared between the two groups, as detailed in Table 5.

**TABLE 5:** Comparison of pulmonary bullae characteristics in patients of different body types.

Characteristics	BMI		*F*/χ^2^/*Z*	P
	BMI < 23.03 kg/m^2^ (BMI<23.03 kg/cm^2^; *n* = 232)	BMI ≥ 23.03 kg/m^2^ (BMI ≥ 23.03 kg/cm^2^; *n* = 235)		
Gender				
Male	199(85.78%)	189(80.43%)	2.378	0.139
Female	33(14.22%)	46(19.57%)		
Age (years)	66(59–72)	62(56–69)	−2.218	0.027
Classification				
Bullous emphysema	133(57.33%)	78(33.19%)	29.293	< 0.001
Intrapleural pulmonary bullae	41(17.67%)	79(33.62%)		
Subpleural pulmonary bullae	58(25.00%)	78(33.19%)		
Maximum bulla size				
Long diameter (mm)	22.80(16.10–39.50)	20.00(14.70–29.90)	−2.808 −2.409 −2.641	0.005 0.016 0.008
Short diameter (mm)	16.30(11.90–24.30)	14.40(11.00–20.20)		
Surface area (mm^2^)	301.19(154.06–735.48)	238.80(124.82–459.44)		
Number of pulmonary bullae				
1	57(24.57%)	85(36.17%)	18.706	< 0.001
2–5	16(6.90%)	34(14.47%)		
>5	159(68.53%)	116(49.36%)		
Regular edges				
Yes	85(36.64%)	126(53.62%)	13.589	< 0.001
No	147(63.36%)	109(46.38%)		
Presence or absence of septa within the bullae				
Yes	171(73.71%)	125(53.19%)	21.171	< 0.001
No	61(26.29%)	110(46.81%)		
Presence of thick-walled bullae (wall thickness > 1mm)				
Yes	124(53.45%)	97(41.28%)	6.938	0.009
No	108(46.55%)	138(58.72%)		

F, ANOVA statistic; χ^2^, Chi-square value; U: Mann-Whitney U test.

The statistical results showed that patients in the low BMI group were significantly older than those in the high BMI group (*P* < 0.05). No statistically significant difference was observed in gender distribution between the two groups (*P* > 0.05). Significant differences were found in the distribution of pulmonary bullae phenotypes and the number of bullae between the two groups (*P* < 0.05).

Patients in the low BMI group demonstrated significantly greater maximum bulla length, maximum bulla width, and surface area compared to those in the high BMI group (*P* < 0.05). The proportion of patients with “internal septations” and “thick-walled” bullae was significantly higher in the low BMI group than in the high BMI group (*P* < 0.05).

The proportion of patients with “regular margins” was significantly higher in the high BMI group compared to the low BMI group (*P* < 0.05).

### 3.4 Comparison of pulmonary function parameters among different phenotypes of pulmonary bullae

This section included a total of 201 patients with pulmonary bullae who underwent pulmonary function testing. The collected pulmonary function parameters included FVC, FVC% predicted, FEV_1_, FEV_1_/FVC ratio, FEV_1_% predicted, and D_*L*_CO% predicted. Patients were classified into three groups based on bullae morphology: bullous emphysema, intrapulmonary bullae, and subpleural bullae. Detailed comparisons among the three groups are presented in Table 6.

**TABLE 6:** Comparison of pulmonary function indicators among patients with different subtypes of pulmonary bullae.

Characteristics	Classification			*F/H*	*P*
	Bullous emphysema (*n* = 110)	Intrapleural pulmonary bullae (*n* = 37)	Subpleural pulmonary bullae (*n* = 54)		
FVC	3.23 ± 0.79	3.36 ± 0.86	3.67 ± 0.92^a^	4.391	0.014
FVC%pred	90.48 ± 20.43	103.09 ± 17.84^a^	95.51 ± 18.68	5.503	0.005
FEV_1_	1.75 ± 0.77	2.30 ± 0.71^a^	2.72 ± 0.81^ab^	28.073	< 0.001
FEV_1_%pred	59.88(39.80–84.39)	92.71(75.10–107.27)^c^	88.05(78.93–105.09)^c^	39.506	< 0.001
FEV_1_/FVC	63.58(48.16–76.94)	84.64(77.41–91.69)^c^	89.91(79.82–96.64)^c^	67.814	< 0.001
D_*L*_CO%pred	54.19 ± 24.15	83.45 ± 17.43^a^	71.75 ± 24.15^ab^	25.947	< 0.001

^a^Indicates that, following the LSD-*t*-test between groups, *P*^a^ < 0.05 when compared with bullous emphysema.

^b^Indicates that, following the LSD–*t*-test between groups, *P*^b^ < 0.05 when compared with intrapleural pulmonary bullae.

^c^Indicates that, after the independent—sample median test with Bonferroni correction between groups, *P*^c^ < 0.05 when compared with bullous emphysema. F, ANOVA statistic; H, Kruskal-Wallis H statistic.

The bullous emphysema group exhibited significantly lower FVC compared to the subpleural bullae group (*P* < 0.05), and significantly lower FVC% predicted compared to the intrapleural pulmonary bullae group (*P* < 0.05).

The bullous emphysema group demonstrated significantly lower FEV_1_, FEV_1_% predicted, FEV_1_/FVC ratio, and D_*L*_CO%pred compared to the other two groups (*P* < 0.05).

The subpleural bullae group showed significantly higher FEV_1_ but significantly lower D_*L*_CO%pred compared to the intrapulmonary bullae group (*P* < 0.05).

### 3.5 Analysis of pulmonary bullae characteristics in patients with COPD

Based on pulmonary function test results, patients with a post-bronchodilator FEV_1_/FVC ratio < 0.70 were classified into the COPD group (*n* = 83) and the non-COPD group (*n* = 118). Baseline characteristics and morphological features of pulmonary bullae were compared between the two groups, as detailed in Table 7.

**TABLE 7:** Analysis of pulmonary bullae characteristics in patients with COPD.

Characteristics	COPD group (*n* = 83)	Non-COPD group (*n* = 118)	*F*/χ^2^/U	*P*
Gender			1.942	0.163
Male	78(94.00%)	104(88.10%)		
Female	5(6.00%)	14(11.9%)		
Age	64(60–69)	64.5(56.75–70.00)	−0.250	0.802
BMI (kg/m^2^)	21.99 ± 3.44	23.03 ± 3.32	2.144	0.033
Maximum bulla size				
Long diameter (mm)	31.60(22.10–51.90)	20.50(15.93–31.45)	−5.008	<0.001
Short diameter (mm)	20.00(14.60–30.60)	14.90(11.48–19.90)	−4.709	<0.001
Surface area (mm^2^)	509.97(294.94–1210.95)	236.76(155.66–459.15)	−5.195	<0.001
Number of pulmonary bullae			22.626	<0.001
1	4(4.80%)	32(27.10%)		
2–5	2(2.40%)	11(9.30%)		
>5	77(92.80%)	75(63.60%)		
Classification			31.833	<0.001
Bullous emphysema	65(78.30%)	45(38.10%)		
Intrapleural pulmonary bullae	8(9.60%)	29(24.60%)		
Subpleural pulmonary bullae	10(37.30%)	44(37.30%)		
Regular edges			11.020	0.001
Yes	19(22.90%)	54(45.80%)		
No	64(77.10%)	64(54.20)		
Presence or absence of septa within the bullae			12.448	<0.001
Yes	10(12.00%)	40(33.90%)		
No	73(88.00%)	78(66.10%)		

F, ANOVA statistic; χ^2^, Chi-square value; U, Mann-Whitney U test.

As shown in the Table 7, no statistically significant differences were observed in baseline characteristics such as gender distribution and age between the COPD group and the non-COPD group (*P* > 0.05). However, patients in the COPD group had significantly lower BMI compared to those in the non-COPD group (*P* < 0.05).

The maximum bulla length, maximum bulla width, and surface area were significantly greater in the COPD group than in the non-COPD group (*P* < 0.05). Additionally, significant differences were found between the two groups in terms of the distribution of bullae and type (*P* < 0.05).

The proportions of patients with “regular margins” and “internal septations” were significantly lower in the COPD group compared to the non-COPD group (*P* < 0.05).

## 4 Discussion

Pulmonary bullae are air-filled cystic spaces formed by the local rupture and coalescence of alveolar walls. Based on lesion location, extent, and pathological morphology, they can be classified into subtypes including bullous emphysema, intrapulmonary bullae, and subpleural bullae, which may differ in pathogenesis, clinical progression, and prognosis. This study enrolled 467 patients with pulmonary bullae and systematically compared demographic characteristics, bulla morphology, and pulmonary function parameters among the three subtypes, aiming to elucidate their clinical features and underlying pathophysiological mechanisms to inform clinical management.

The cohort predominantly comprised middle-aged and elderly males (median age: 64 years; male: 83.1%), with 46.7% having a smoking history. Significant intergroup differences were observed in gender, age, BMI, and smoking history (*P* < 0.05). Patients with bullous emphysema were older, had lower BMI, and exhibited a higher smoking rate (60.66%), suggesting a strong association with cumulative lung injury from smoking and aging (4). In contrast, patients with intrapulmonary and subpleural bullae were generally younger with normal BMI, potentially related to localized developmental abnormalities or aberrant repair following acute lung injury, with weaker correlations to age and smoking. Notably, pulmonary comorbidities were frequent and overlapping, with emphysema being the most common (56.0%), reinforcing their shared pathological basis. The high detection rate of pulmonary nodules (39.27%) may reflect both common risk factors such as smoking and the increased sensitivity of CT screening. The substantial prevalence of extrapulmonary comorbidities—including hypertension, coronary heart disease, and diabetes—suggests that pulmonary bullae are not merely a localized pulmonary condition but also a manifestation of systemic aging and smoking-related damage, necessitating comprehensive patient management.

Regarding morphological characteristics, patients with bullous emphysema demonstrated significantly greater maximum bulla length, width, and surface area compared to the other two groups (*P* < 0.05), and the subpleural bullae group exhibited significantly greater maximum length than the intrapulmonary group (*P* < 0.05). These findings reflect the diffuse pathology of bullous emphysema: long-term exposure to harmful stimuli such as cigarette smoke induces chronic inflammation in distal airspaces, leading to alveolar wall destruction, extracellular matrix degradation (10–12), and progressive airspace coalescence into larger bullae. Further analysis identified smoking as a critical factor promoting bulla enlargement and morphological irregularity. The smoking group showed significantly greater bulla dimensions, higher proportions of internal septations and thick walls, and lower proportions of regular margins (*P* < 0.05). The underlying mechanisms involve smoking-induced chronic inflammation upregulating matrix metalloproteinases (including neutrophil elastase, MMP-2, and MMP-9) (13), disrupting the protease-antiprotease balance and causing excessive degradation of alveolar structural components. Additionally, harmful components in cigarette smoke directly damage type II alveolar epithelial cells (14), impairing alveolar wall repair and accelerating structural destruction, resulting in irregular bulla coalescence.

BMI also significantly influenced bulla morphology. When stratified by median BMI (23.03 kg/m^2^), the low BMI group exhibited larger bullae with more frequent septations and thick walls, and fewer regular margins (*P* < 0.05). Reduced chest wall fat and muscle mass in low-BMI patients may decrease parenchymal traction, leading to uneven stress distribution and localized overexpansion of alveolar walls. Furthermore, low BMI often indicates malnutrition, which can precipitate respiratory muscle atrophy and increased infection susceptibility (15–17). Conversely, adiponectin secreted by adipose tissue in high-BMI patients negatively correlates with MMP-9 levels (18), potentially attenuating alveolar destruction by reducing MMP-9 activity and thus inhibiting excessive bulla coalescence. These findings highlight smoking and low BMI as two key modifiable factors promoting pulmonary bulla progression.

Pulmonary function analysis revealed significant heterogeneity in functional impairment across subtypes. Patients with bullous emphysema had significantly lower FEV_1_, FEV_1_% predicted, FEV_1_/FVC ratio, and DLCO compared to the other groups (*P* < 0.05), indicating more severe obstructive ventilatory dysfunction and diffusion impairment. This is attributable to diffuse alveolar wall destruction in bullous emphysema, which reduces lung elastic recoil and decreases the effective surface area for gas exchange. In contrast, intrapulmonary and subpleural bullae, being localized lesions, had relatively limited impact on global pulmonary function. However, differences emerged between these two localized subtypes: the subpleural group showed significantly higher FEV_1_ but lower DLCO than the intrapulmonary group (*P* < 0.05), likely due to anatomical location—subpleural bullae exert less compression on airways, preserving ventilation, while intrapulmonary bullae cause less disruption to local alveolar exchange areas, better preserving diffusion capacity.

When patients were stratified by COPD status based on pulmonary function testing, the COPD group demonstrated significantly greater maximum bulla length, width, and surface area, with a lower proportion of internal septations compared to the non-COPD group (*P* < 0.05). This finding reflects the diffuse lung destruction characteristic of COPD, where protease-antiprotease imbalance and oxidative stress drive widespread alveolar wall degradation (4), promoting progressive bulla coalescence. In contrast, bullae in non-COPD patients are typically localized lesions without this diffuse destructive background, resulting in smaller bullae that may retain multilocular septated structures. These results suggest that the presence of large, irregular pulmonary bullae on CT images should raise clinical suspicion of underlying COPD, warranting confirmatory pulmonary function testing.

These findings have multiple clinical implications. First, CT-based phenotyping of pulmonary bullae can help predict the degree of functional impairment: patients with bullous emphysema require close monitoring of pulmonary function and surveillance for complications such as pneumothorax (19) and respiratory failure, while those with localized bullae may be managed with extended follow-up intervals. Second, smoking and low BMI are modifiable risk factors for bulla progression; smoking cessation should be strongly emphasized, and nutritional support with pulmonary rehabilitation should be considered for underweight patients to potentially slow disease progression (15–17, 20, 21). Third, in patients with concurrent COPD, management should integrate comprehensive COPD care (e.g., bronchodilators, pulmonary rehabilitation) with monitoring for bulla-related complications. Fourth, low-BMI patients should receive pneumococcal and influenza vaccinations to reduce infection-triggered exacerbations, and all patients should be counseled to avoid activities that may precipitate bulla rupture, such as scuba diving and high-altitude flying.

This study has several limitations. First, the cross-sectional design precludes causal inferences. Second, detailed data on COPD duration, severity, and pharmacological treatment were unavailable, limiting in-depth analysis. Third, α1-antitrypsin levels were not measured, preventing exclusion of α1-antitrypsin deficiency as a contributing factor. Fourth, smoking history was treated as a binary variable, precluding assessment of dose-response relationships. Future longitudinal studies are warranted to investigate the dynamic evolution of pulmonary bullae and their prognostic significance.

This study proposes a refined CT-based phenotypic classification of pulmonary bullae and elucidates its functional implications. Moving beyond traditional generalized conceptualizations, we characterized three distinct phenotypes—bullous emphysema, intraparenchymal bullae, and subpleural bullae—based on detailed imaging features and systematically compared their morphological characteristics and pulmonary function profiles. Our findings demonstrate that bullous emphysema not only exhibits significantly larger bulla volumes but is also associated with more severe pulmonary function impairment, providing direct evidence to inform personalized clinical follow-up and intervention strategies tailored to different bulla phenotypes.

## Data Availability

The raw data supporting the conclusions of this article will be made available by the authors, without undue reservation.

## References

[1] Hansell DM Bankier AA MacMahon H McLoud TC Müller NL Remy J. Fleischner Society: glossary of terms for thoracic imaging. *Radiology.* (2008) 246:697–722. 10.1148/radiol.2462070712 18195376

[2] Hyun K Kim JJ Im KS Han SC Ryu JH. Visual and predictive assessment of pneumothorax recurrence in adolescents using machine learning on chest CT. *J Clin Med.* (2025) 14:5956. 10.3390/jcm14175956 40943716 PMC12428816

[3] Riveiro-Blanco V Pou-Álvarez C Ferreiro L Toubes ME Quiroga-Martínez J Suárez-Antelo J et al. Recurrence of primary spontaneous pneumothorax: associated factors. *Pulmonology.* (2022) 28:276–83. 10.1016/j.pulmoe.2020.06.003 32601016

[4] Siddiqui NA Sankari A Mansour MK Nookala V. Bullous Emphysema. *StatPearls.* Treasure Island (FL): StatPearls Publishing (2025). 30725928

[5] Stern EJ Webb WR Weinacker A Müller NL. Idiopathic giant bullous emphysema (vanishing lung syndrome): imaging findings in nine patients. *AJR Am J Roentgenol.* (1994) 162:279–82. 10.2214/ajr.162.2.8310909 8310909

[6] Wu MC Wu ZD. *Huang Jiasi’s Surgery.* 8th ed. The People’s Health Press Co., Ltd (2020).

[7] Lee KC Kang EY Yong HS Kim C Lee KY Hwang SH et al. A stepwise diagnostic approach to cystic lung diseases for radiologists. *Korean J Radiol.* (2019) 20:1368–80. 10.3348/kjr.2019.0057 31464115 PMC6715565

[8] Raoof S Bondalapati P Vydyula R Ryu JH Gupta N Raoof S et al. Cystic lung diseases: algorithmic approach. *Chest.* (2016) 150:945–65. 10.1016/j.chest.2016.04.026 27180915 PMC7534033

[9] Remy-Jardin M Remy J Giraud F Wattinne L Gosselin B. Computed tomography assessment of ground-glass opacity: semiology and significance. *J Thorac Imaging.* (1993) 8:249–64. 10.1097/00005382-199323000-00001 8246323

[10] Kemp SV Polkey MI Shah PL. The epidemiology, etiology, clinical features, and natural history of emphysema. *Thorac Surg Clin.* (2009) 19:149–58. 10.1016/j.thorsurg.2009.03.003 19662957

[11] Liu G Hsu AC Geirnaert S Cong C Nair PM Shen S et al. Vitronectin regulates lung tissue remodeling and emphysema in chronic obstructive pulmonary disease. *Mol Ther.* (2025) 33:917–32. 10.1016/j.ymthe.2025.01.032 39838644 PMC11897773

[12] Tuder RM Petrache I. Pathogenesis of chronic obstructive pulmonary disease. *J Clin Invest.* (2012) 122:2749–55. 10.1172/jci60324 22850885 PMC3408733

[13] Ghosh A Coakley RD Ghio AJ Muhlebach MS Esther CR Jr. Alexis NE et al. Chronic E-cigarette use increases neutrophil elastase and matrix metalloprotease levels in the lung. *Am J Respir Crit Care Med.* (2019) 200:1392–401. 10.1164/rccm.201903-0615oc 31390877 PMC6884043

[14] Simborio H Hayek H Kosmider B Elrod JW Bolla S Marchetti N et al. Mitochondrial dysfunction and impaired DNA damage repair through PICT1 dysregulation in alveolar type II cells in emphysema. *Cell Commun Signal.* (2024) 22:562. 10.1186/s12964-024-01896-0 39578839 PMC11583753

[15] Li J Zhu L Wei Y Lv J Guo Y Bian Z et al. Association between adiposity measures and COPD risk in Chinese adults. *Eur Respir J.* (2020) 55:1901899. 10.1183/13993003.01899-2019 31980495 PMC7236866

[16] Charususin N Dacha S Gosselink R Decramer M Von Leupoldt A Reijnders T et al. Respiratory muscle function and exercise limitation in patients with chronic obstructive pulmonary disease: a review. *Expert Rev Respir Med.* (2018) 12:67–79. 10.1080/17476348.2018.1398084 29072087

[17] Lee SJ Kim SW Kong KA Ryu YJ Lee JH Chang JH. Risk factors for chronic obstructive pulmonary disease among never-smokers in Korea. *Int J Chron Obstruct Pulmon Dis.* (2015) 10:497–506. 10.2147/copd.s77662 25784796 PMC4356706

[18] Liu F Zhu L. [Expression of adiponectin in non-small cell lung cancer and its relationship with MMP-9 and angiogenesis]. *Zhong Nan Da Xue Xue Bao Yi Xue Ban.* (2015) 40:579–84. 10.11817/j.issn.1672-7347.2015.06.002 26164504

[19] Desai P Steiner R. Images in COPD: giant bullous emphysema. *Chronic Obstr Pulm Dis.* (2016) 3:698–701. 10.15326/jcopdf.3.3.2016.0154 28848895 PMC5556766

[20] Holtjer JCS Bloemsma LD Beijers R Cornelissen MEB Hilvering B Houweling L et al. Identifying risk factors for COPD and adult-onset asthma: an umbrella review. *Eur Respir Rev.* (2023) 32:230009. 10.1183/16000617.0009-2023 37137510 PMC10155046

[21] Zhang X Chen H Gu K Chen J Jiang X. Association of body mass index with risk of chronic obstructive pulmonary disease: a systematic review and meta-analysis. *Copd.* (2021) 18:101–13. 10.1080/15412555.2021.1884213 33590791

